# Supplementary material for: Global musical diversity is largely independent of linguistic and genetic histories
Source: Nat Commun. 2024 May 10;15:3964. doi: 10.1038/s41467-024-48113-7 (PMC11087526; doi:10.1038/s41467-024-48113-7)
Supplement: Supplementary file 1 — Supplementary Information [file 41467_2024_48113_MOESM1_ESM.pdf]

Supplementary Information for: Passmore et. al, Independent histories underlie global musical, linguistic, and genetic diversity.

Supplementary Note 1: Cantometrics Data Processing

S1.1 Musical Data sources

Data for this project is drawn from the Cantometrics dataset of the Global Jukebox [1]. Cantometrics codes 5,778 songs from 992 societies on 37 different variables. Historically, each variable was referred to as a *Line*. We will use Line to refer to each variable moving forward to ensure our analyses can be aligned with existing Cantometrics work (E.g. Line 1 refers to the first variable).

The nature of Cantometrics means that all songs contain information on all variables. However, there are a small number of cases where this is not the case, which may have been caused by illegible handwriting within the original paper documents, or a copying error. We remove 300 songs that do not have complete codes for all 37 Lines.

Songs can display multiple characteristics within a Cantometric Line throughout the performance, meaning some songs can have multiple codings for any particular variable. For analytical reasons, we require one value per song, per variable which we select at random. This affects 3% of the dataset.

Table S1: Description of all Cantometric variables from the Global Jukebox Database. This table as well as the original data, and descriptions of each variable's codes, can be found at https://github.com/theglobaljukebox/cantometrics. Detailed descriptions of coding criteria, along with audio examples, can be accessed at http://theglobaljukebox.org in the “Songs of Earth” section.

**Line**	**Description**	**Range**
Line 1	The social organization of the vocal group	1 - 13 (no singers - interlocking)
Line 2	Relationship of orchestra to vocal parts	1 - 13 (no instruments - large orchestra)
Line 3	Social organization of the orchestra	1 - 13 (no instruments - overlapping orchestral groups)
Line 4	Musical organization of the vocal part	1 - 5 (monophony - polyphony)
Line 5	Tonal blend of the vocal group	1 - 5 (solo - max. blend)
Line 6	Rhythmic coordination of the vocal group	1 - 5 (solo - max. coordination)
Line 7	Musical organization of the orchestra	1 - 5 (no instruments - polyphony)
Line 8	Tonal blend of the orchestra	1 - 5 (no instruments - max. blend)
Line 9	Rhythmic coordination of the orchestra	1 - 5 (no instruments - max. coordination)
Line 10	Repetition of text	1 - 5 (little or no repetition - extreme repetition)
Line 11	Overall Rhythm: vocal	1 - 6 (no singers - parlando rubato)
Line 12	Rhythmic relationship within the vocal group	1 - 7 (no Rhythm - counterpoint)
Line 13	Overall Rhythm: orchestra	1 - 6 (no instruments - parlando rubato)
Line 14	Rhythmic relationship within the orchestra	1 - 7 (no orchestra - counterpoint)
Line 15	Melodic shape	1 - 4 (arched melody - descending melody)
Line 16	Melodic form	1 -13 (through composed - round form)
Line 17	Phrase length	1 - 5 (long phrases - short phrases)
Line 18	Number of phrases	1 - 13 (eight phrases before repeat - one or two phrases)
Line 19	Position of final tone	1 - 5 (final note is lowest - final note is highest)
Line 20	Melodic range	1 - 5 (monotone to major second - two octaves or more)
Line 21	Interval size	1 - 5 (monotone - very large intervals)
Line 22	Polyphonic type	1 - 6 (no polyphony - counterpoint)
Line 23	Embellishment	1 - 5 (extreme embellishment - little or no embellishment)

Line 24	Tempo	1 - 6 (very slow - very fast)
Line 25	Volume	1 - 5 (very soft - very loud)
Line 26	Rubato: vocal	1 - 4 (extreme rubato - no rubato)
Line 27	Rubato: orchestra	1 - 4 (extreme rubato - no rubato)
Line 28	Glissando	1 - 4 (max. glissando - little or no glissando)
Line 29	Melisma	1 - 3 (much melisma - little or no melisma)
Line 30	Tremolo	1 - 5 (much tremolo - little or no tremolo)
Line 31	Glottal	1 - 5 (much glottal - little or no glottal)
Line 32	Vocal pitch (register)	1 - 5 (very high - very low)
Line 33	Vocal width	1 - 6 (very narrow - yodel)
Line 34	Nasality	1 - 5 (extreme nasalisation - little or no nasalisation)
Line 35	Rasp	1 - 5 (extreme rasp - no rasp)
Line 36	Accent	1 - 5 (forceful accent - relaxed)
Line 37	Enunciation	1 - 5 (precise enunciation - soft enunciation)

S1.2 Standardization

All Cantometrics Lines were originally recorded on a scale with a maximum value of 13. Each Cantometrics variable had a different number of possible responses, meaning this scale was not spaced equally across variables. We rescale all variables in Cantometrics to be between 0 and 1. To do this, each variable is rescaled from the 13-point scale to a linearly increasing scale. For example: Line 5 (Tonal blend of the vocal group) had 5 codes 1, 4, 7, 10, 13, which are respectively coded to 1, 2, 3, 4, 5. Then, the linear scale is standardized using the following formula:

$$(code - 1) / 13 - 1 \qquad \text{(S1)}$$

Subtracting 1 from the code and maximum value means the rescaled variables start at 0.

S1.4 Rescaling

We reverse the codings of several existing Cantometric variables so that all variables align high values with a more frequent occurrence of what the variable measures. These are listed in table S2. Codes are reversed by subtracting the standardized scores from 1.

Table S2. List of Lines where high values indicate less of a musical feature. This table shows the high and low values on the original scale, which are reversed in our analyses.

**Line**	**Original Codings**	
	Code = 1	*Code = 13*
Line 17: Phrase Length	Very long phrases.	Very short phrases. (1-2 seconds).
Line 18: Melodic form	There are more than eight phrases before a full repeat.	One or two phrases, symmetrically arranged
Line 23: Embellishment	Extreme embellishment.	Little or no embellishment.
Line 26: Vocal Rubato	Extreme rubato.	No rubato
Line 27: Orchestral Rubato	Extreme rubato.	No rubato
Line 28: Glissando	Maximal glissando.	Little or no glissando.
Line 29: Melisma	Much melisma.	Little or no melisma.
Line 30: Tremolo	Much tremolo.	Little or no tremolo.
Line 31: Glottal	Much glottal.	Little or no glottal.
Line 32: Vocal Pitch	Very high.	Very low.
Line 34: Nasality	Extreme nasalization.	Little or no nasalization.
Line 35: Rasp	Extreme rasp	None. Voices lack rasp.
Line 36: Accent	Very forceful accent.	Very relaxed, absence of clear stresses.
Line 37: Enunciation	Very precise enunciation.	Very softened enunciation.

S1.5 Songs and Societies

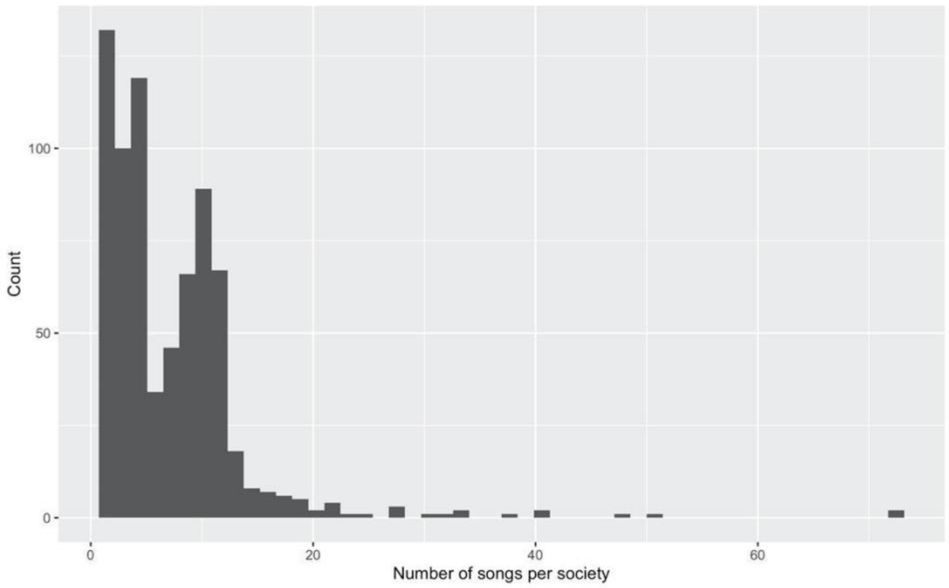

Fig. S1: A histogram showing the distribution of songs per society (N societies = 1,026). Songs are distributed unevenly between societies, with 73 songs being the most any one society has and 232 societies containing only one song.

S1.6 Pairing Cantometrics to Genetics and Language data

To analyze the patterns of between-group differences in musical style, Cantometric societies are paired to both a language and genetic sample. Languages are identified using Glottocodes [2]. The Glottocode is used to link the Cantometric society to a genetic population from the database GeLaTo [3], a global genetic diversity panel annotated for linguistic affiliation, and from other published genetic data (see Supplementary Data S5 for a list of genetic publications used). The Cantometric society is associated with the genetic population either through a perfect Glottocode match, through alternative population name matches, or if no identical match is found through a closely related language. Geographic proximity between the location of a Cantometric society and the genetic population was also considered.

S1.7 Cantometrics Coding Procedure and Reliability

The Cantometric codings in the Global Jukebox have undergone an extensive quality control procedure and testing reliability and accuracy documented in a previous publication [1]. The following quote summarizes the results of these quality controls (see Fig. S8 and Table S12 in Wood et al. for a detailed breakdown of reliability statistics for each variable under different coder conditions and combinations):

> "Overall, our analyses suggest that both coding reliability (mean $\kappa = 0.54$; Fig. S8 and Table S12) and accuracy (approximately 0.4-1% rate of unambiguous coding/data entry errors; Fig. S9) are at acceptable levels on average. However, there was also substantial variation in reliability across variables. Some variables showed near-perfect consensus: for example Line 4 differentiating solo vs. different types of group singing ($\kappa = 0.94$ / 89% agreement) and Line 7 which captures similar information but for instrumental accompaniment ($\kappa = 0.92$ / 86% agreement). Other variables had very low reliability effectively at chance levels (e.g., nasality [Line 33], vocal width [Line 32])." (Wood et al., 2021)

Note also that mean Kappa of 0.54 is much higher than comparable cross-cultural music datasets published in high-profile journals (e.g., mean Kappa of 0.24 in Mehr et al., 2019, *Science [reported in Wood et al. 2022 Supporting Information]*; mean Kappa of 0.45 in Savage et al., 2015, *PNAS Supporting Information*).

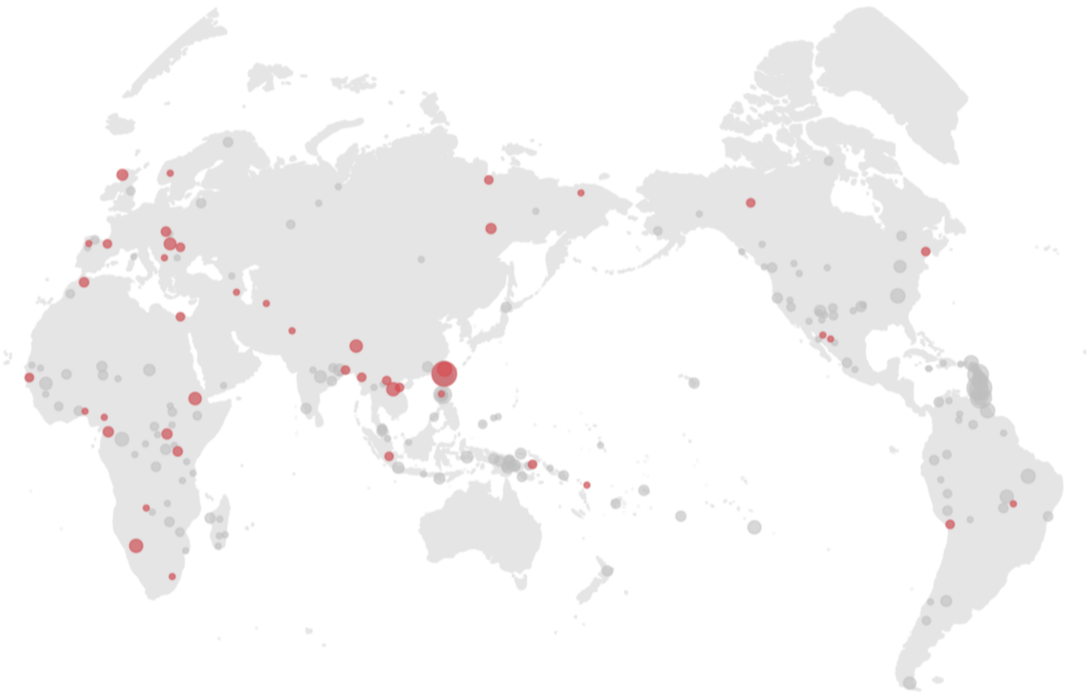

Fig. S2: Map of 222 Cantometrics societies (represented by 3,063 songs). These songs make up the set of societies for which we have ten songs or more. 44 societies are matched to both genetic and linguistic data are indicated in red (societies without matching data are in grey).

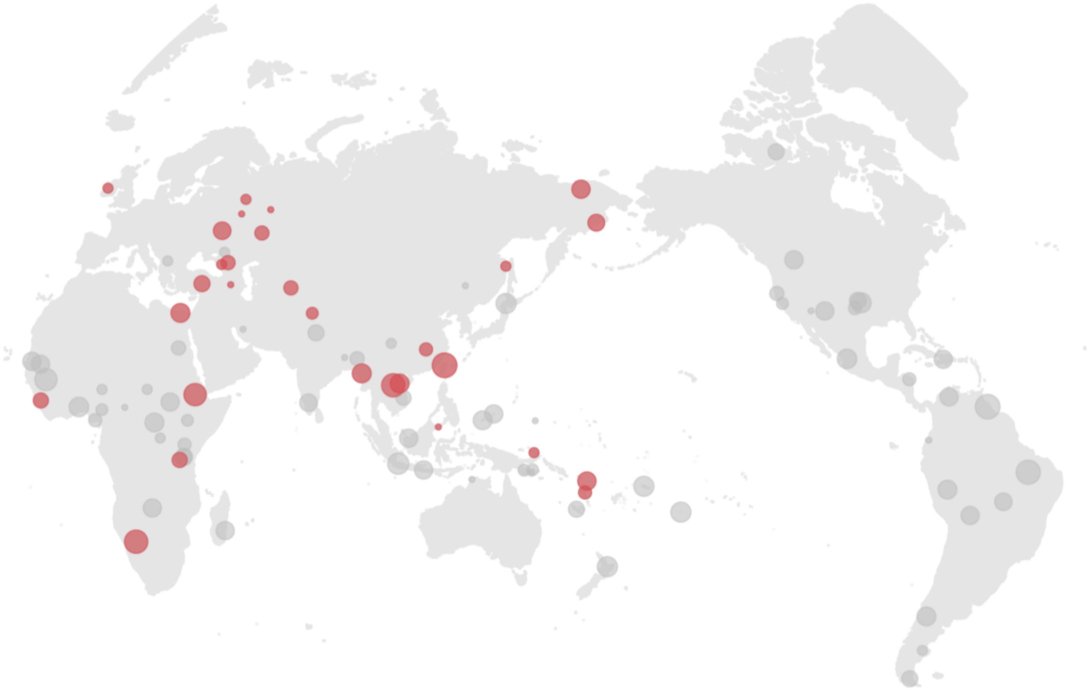

Fig. S3: Map of 95 Cantometrics societies (represented by 689 songs). These songs make up the set of societies which are matched to the SCCS. 21 societies are matched to both genetic and linguistic data are indicated in red (societies without matching data are in grey).

Table S3. Latent variable model results summary

Data	N Soc	N Songs	LV fit	Latent Variable model	Within vs Between Variance	Delta Scores (N = 50)
2 or more songs	719	5,242	RSMEA=0.06; SRMR=0.05; CFI=0.95	Table S9: All correlations > 0.97	Supplementary Data S1 & Figure S5	In main text & Supplementary Data S3 & Fig S10
10 or more songs	222	3,063	RSMEA=0.06; SRMR=0.05; CFI=0.94	Table S9: All correlations > 0.97	Supplementary Data S1 & Figure S5	Supplementary Data S3 & Fig S10. Some variables in some areas show higher levels of tree-likeness
SCCS Sample	95	796	RSMEA=0.06; SRMR=0.05; CFI=0.95	Table S9: All correlations > 0.97	Supplementary Data S1 & Figure S5. SCCS shows higher between group variance.	Supplementary Data S3 & Fig S10
With Restricted Variable set	719	5,242	RSMEA=0.07; SRMR=0.04; CFI=0.95	Table S10: All correlations >0.7 except Tension	-	-

Table S4. Genetic, Linguistic, and Geographic results comparison result summary

Data	N Societies	Geographic Autocorrelation	Mantel Tests	Partial RDA
2 or more songs	117	Figure 3	Supplementary Data S6	Supplementary Data S6
10 or more songs	44	Figure S8	Supplementary Data S7	Supplementary Data S7
SCCS Sample	21	Figure S9. SCCS shows low levels of Autocorrelation	Supplementary Data S8	Supplementary Data S8. SCCS results are not similar to other datasets.

Supplementary Note 2: Latent Variable modeling

S3.1: *Summary of Latent variable model*

Latent variable modelling is performed using lavaan and R v4.1 [4], using a set of 5,242 songs from 719 societies. The model is described as equations in Table S5. This model meets all standard latent variable model statistics: RMSEA = 0.06, SRMR = 0.06, and CFI = 0.93. In addition to the six latent variables, the model estimated the correlation between latent variables and incorporated seven correlations between Cantometric variables which were not explained by the latent variables. The numerical results provided are a completely standardized solution.

Table S5. Equation description of the latent variable model (N songs = 5,242). Showing the description of each Cantometrics and their standardized weight, indicating their contribution to the latent variable.

<u>*Legend*</u><br>*=~ indicates a latent variable*<br>*~~ indicates variables that are correlated*<br>*Numbers before a variable are indicative of the weight of the relationship between that variable and the variable on the left-hand side of the equation.*
Articulation =~ 0.62 * Enunciation +<br>-0.74 * Repetition of text +<br>0.72 * Glottal +<br>0.29 * Embellishment<br><br>Ornamentation =~ 0.57 * Glissando +<br>0.81 * Melisma +<br>0.59 * Embellishment +<br>0.47 * Tremolo<br><br>Rhythm =~ 0.57 * Overall Rhythm: vocal +<br>-0.58 * Tempo +<br>0.56 * Phrase length<br><br>Dynamics =~ 0.74 * Volume +<br>0.77* Tempo +<br>0.28 * Vocal pitch (register)<br><br>Tension =~ 0.70 * Nasality +<br>0.42 * Rasp +<br>-0.68 * Vocal width +<br>-0.39 * Melisma<br><br># Allow latent variables to be correlated<br>Articulation ~~ 0.12 * Ornamentation<br>Articulation ~~ 0.27 * Rhythm<br>Articulation ~~ -0.13 * Dynamics<br>Articulation ~~ 0.34 * Tension<br>Ornamentation ~~ 0.04 * Dynamics<br>Ornamentation ~~ 0.625 * Rhythm<br>Ornamentation ~~ 0.53 * Tension<br>Rhythm ~~ -0.19 * Dynamics<br>Rhythm ~~ -0.15 * Tension<br>Dynamics ~~ 0.17 * Tension<br><br># Highly related variables unexplained by model variance<br>Embellishment ~~ 0.35 * Tremolo<br>Nasality ~~ 0.43 * Rasp<br>Enunciation ~~ 0.27 * Accent<br>Repetition of text ~~ -0.93 * Glottal<br>Vocal pitch (register) ~~ -0.11 * Rasp<br>Phrase length ~~ 0.22 * Vocal width

S3.2: Construction of Latent variable model

The construction of the latent variables is determined using a combination of principal component analysis, and an existing analysis of the Cantometrics dataset. First, we implemented a principal component analysis using all Cantometrics variables, replicating the process used in early Cantometrics work, although expanding from the initial subset of ~1,800 songs to 5,242 songs [5]. Through this process we identify a subset of Cantometrics variables which contain dependencies for songs that involve either solo singers or solo instrumentalists (table S4 & S5). For example: if a song has a solo singer (Line 1 code 2 or 3), it must also have no tonal blend (Line 5 code 1). These dependencies meant the first two principal components effectively differentiate between songs with group or solo instrumental performances (PC1) and group or solo vocal performances (PC2). This is visualized in figure S2. There are two solutions to this - either analyze all songs excluding all but one of the dependent variables in table S4 and S5, or exclude all songs which contain solo instrumentalists or songs. We decide the former is a better solution for studying global musical diversity.

Table S6: Table of dependencies in vocal variables. Described as the Line number, and the code for that line that the dependency occurs on after a colon. *Note: Line 1: 1 (No Singers) cannot occur in this dataset, although it does exist in the coding scheme – all songs must have singers.

**Vocal dependencies**	
Line 1: (code) 2 & 3	Line 4: 4
Line 1: 2 & 3	Line 5: 1
Line 1: 2 & 3	Line 6: 1
Line 1: 2 & 3	Line 12: 1
Line 1: 2 & 3	Line 22: 1
Line 1: 1*	Line 11: 1
Line 11: 13	Line 26: 1

Table S7: Table of dependencies in orchestral variables. Described as the Line number, and the code for that line that the dependency occurs on after a colon.

**Orchestral dependencies**	
Line 2: (code) 1	Line 3: 1
Line 2: 1	Line 7: 1 & Line 7: 4
Line 2: 1	Line 8: 1
Line 3: 1 & 2	Line 8: 1
Line 2:1	Line 9: 1
Line 3: 1 & 2	Line 9: 1
Line 2: 1	Line 13: 1
Line 3: 1 & 2	Line 13: 1
Line 2:1	Line 14: 1
Line 3: 1 & 2	Line 14: 1
Line 2: 1	Line 27: 13
Line 3: 1 & 2	Line 27: 13

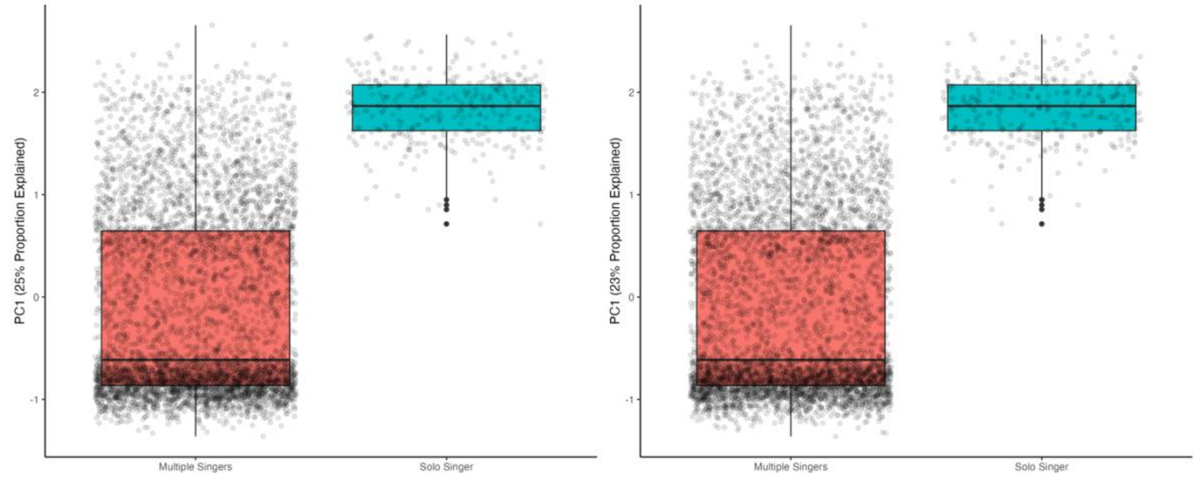

Fig. S4: These plots display the first and second principal components from the Cantometrics dataset using all variables and all songs from societies with two or more songs (n = 5,474). When using all variables, the first and second principal components consist primarily of the dependencies described in table S5 and S6. We plot the principal components based on two categorical variables: Line 2: code 1 to distinguish songs with one or no instruments to songs with multiple instruments and Line 1: code 1, 2, and 3 to distinguish songs where one singer is heard at a time to songs with concurrent singers. We do not use variables that contain dependencies as a result.

Table S8: Variable weights for Principal component and latent variable models for the 2-song dataset (N = 5,242). Principal components consider the weight of all variables on the latent dimensions. Latent variables are designed using a combination of theoretical knowledge of global music, the results of existing analyses of the dataset and the weightings in the principal component analysis.

Line	Description	RC1	Articulation	RC2	Tension	RC3	Ornamentation	Rhythm	RC4	RC5	Dynamics
Line 10	Repetition of text	0.9	-0.8	-0.01		-0.12			-0.14	0.08	
Line 11	Overall Rhythm: vocal	0.01		0.02		-0.51		0.55	-0.14	0.12	
Line 15	Melodic shape	0		-0.03		0.03			-0.14	-0.08	
Line 16	Melodic form	0.2		-0.09		-0.09			-0.87	-0.02	
Line 17	Phrase length	0.23		0.08		-0.47		0.57	-0.12	0	
Line 18	Number of phrases	-0.16		0.06		0.07			0.88	0.01	
Line 19	Position of final tone	0.05		-0.12		0.02			0.08	-0.08	
Line 20	Melodic range	0.14		-0.18		0.22			0.51	0.04	
Line 21	Interval size	0.48		-0.15		-0.21			0.2	-0.1	
Line 23	Embellishment	-0.32	0.28	0.28		0.64	0.61		0.08	0.13	
Line 24	Tempo	-0.08		-0.07		0.52		-0.59	0.11	-0.3	
Line 25	Volume	0.11		0.02		0.11			0.06	0.8	0.86
Line 28	Glissando	0		0.18		0.66	0.56		0.01	-0.02	
Line 29	Melisma	-0.03		-0.09	-0.36	0.75	0.82		-0.01	0.01	
Line 30	Glottal	-0.9	0.79	0.01		0.13			0.14	-0.08	
Line 31	Tremolo	0.09		0.33		0.52	0.44		0.1	0.18	
Line 32	Vocal pitch (register)	-0.12		-0.14		0.03			-0.13	0.56	0.27
Line 33	Vocal width	0.27		-0.61	-0.71	-0.13			-0.03	-0.08	
Line 34	Nasality	-0.11		0.83	0.71	0.15			0	0.03	
Line 35	Rasp	0.07		0.78	0.44	0.01			-0.02	0.08	
Line 36	Accent	0.04		0.22		-0.11			0.11	0.75	0.64
Line 37	Enunciation	-0.68	0.62	0.14		-0.14			0.14	0.04	

S3.3 Conceptualizing Latent dimensions

To help conceptualize the meaning of each dimension, we describe the extremes of each dimension with audio examples. All songs are available at https://theglobaljukebox.org/.

Articulation: Songs that score highly on Articulation contain precise enunciation of non-repeating lyrics (*Example song: Sundanese Song 1* by Javanese performers; song 1562*)*, whereas low scoring songs frequently repeat text with slurred enunciation (*Mens' Chorus 2*, by Dani performers; song 986).

Ornamentation: Songs with high levels of Ornamentation show lots of vocal embellishment, tremolo, or melisma (*Esashi Oiwake* by Hokkaido Japanese performers: song 364), whereas singers in low Ornamentation songs use steady and consistent notes (*Zavan* by Ouldeme performers: song 30146).

Rhythm: Songs that score low on Rhythm have slow, irregular meters and long phrases (*Caravan Bells and the Song of the teamsters* by Tibetan performers, song 398, C in figure 1 in the main text), whereas songs that score high on Rhythm have a fast tempo, and regular meter, and short phrases, as exemplified by the Mbuti song *Alima* (song 9260, D in figure 1).

Dynamics: Songs that score high on Dynamics are loud and intense (*Dance with Long Horns* by Khattak performers, song 749), whereas scoring low indicates a soft song, such as *Efalachid Gelat,* a lullaby-love song from the Ulithi Atoll (song 2628).

Tension: Songs with high Tension have singers that use very nasal, raspy, and constrained voices (*Song with a Xylophone,* by Burmese performers; song 2559, A in figure 1). On the other end of the spectrum is low Tension songs, where singing sounds more relaxed and 'open', as in the Mbendjele song *Djokobo* (song 30063, B in figure 1).

S3.3 Latent variable validity testing

S3.3.1 Restricted song sample comparison

Table S9: Pearson's Correlations between latent variables built using a dataset containing a minimum of two songs per society (5,242 songs and 719 societies), and ten songs per society (3,039 songs and 220 societies), and the Standard Cross-Cultural Sample (724 songs and 110 societies). All variables show significant and strong correlations. The N value for each correlation is the minimum N of the two datasets being compared.

Latent Variable	Datasets	Pearson Correlation	P-value
Articulation	2 vs 10	0.996	<1x10^-16
Articulation	2 vs SCCS	0.999	<1x10^-16
Articulation	10 vs SCCS	0.998	<1x10^-16
Ornamentation	2 vs 10	0.999	<1x10^-16
Ornamentation	2 vs SCCS	0.999	<1x10^-16
Ornamentation	10 vs SCCS	0.986	<1x10^-16
Rhythm	2 vs 10	0.978	<1x10^-16
Rhythm	2 vs SCCS	0.99	<1x10^-16
Rhythm	10 vs SCCS	0.999	<1x10^-16
Dynamics	2 vs 10	0.988	<1x10^-16
Dynamics	2 vs SCCS	0.994	<1x10^-16
Dynamics	10 vs SCCS	0.982	<1x10^-16
Tension	2 vs 10	0.99	<1x10^-16
Tension	2 vs SCCS	0.998	<1x10^-16
Tension	10 vs SCCS	0.99	<1x10^-16

S3.3.1 Restricted variable testing

Table S10: Pearson's Correlations between latent variables built using the full latent variable model and only variables with high reliability (N songs = 5,242). High reliability is determined by having a Cohen's Kappa value greater than .4, which some have proposed as a minimum acceptable level of reliability (e.g., in clinical contexts; Sim & Wright, 2005). The removed variables are Line 17, 24, 28, 30, 33, 34. Line 31 shows low reliability, but removing this variable meant the model did not converge, so it remains. Comparisons were performed using the two-song per society dataset. In two instances, Rhythm and Tension, this left only one variable and so the latent variable was compared to that remaining variable. Excluding Tension, all variables correlate highly regardless of whether using the full or high inter-rater reliability model. Results involving Tension should be interpreted cautiously.

**Full model**	**Restricted model**	**Pearson Correlation**	**P value**
Articulation	Articulation	0.99	<1x10^-16
Ornamentation	Ornamentation	0.82	<1x10^-16
Rhythm	Line 11	0.7	<1x10^-16
Dynamics	Dynamics	0.9	<1x10^-16
Tension	Line 29	0.1	<1x10^-12

Supplementary Note 3: AMOVA & Phi$_{ST}$ analysis

AMOVA analysis is performed using R v4.1 and ade4 v1.7-18 [6]. Information on language family and the geographic Region categorization are taken from Cantometrics metadata.

Musical Phi$_{ST}$ matrices are created using the pairPhiST function within the haplotypes R package [7]. To create the Phi$_{ST}$ matrices for each musical dimension, we calculate the distance between societies using only the variables relating that dimension. To calculate the aggregate musical distance, we calculate the distance between societies using all Cantometrics variables. See the recipe *Phi$_{ST}$* in the *Makefile* for details.

S4.1 Data

Language family and the geographic Region categorisation are taken from Cantometrics metadata. Distances between songs are calculated between songs using Euclidean distance.

Supplementary Data S1 can be found in the Supplementary Tables file.

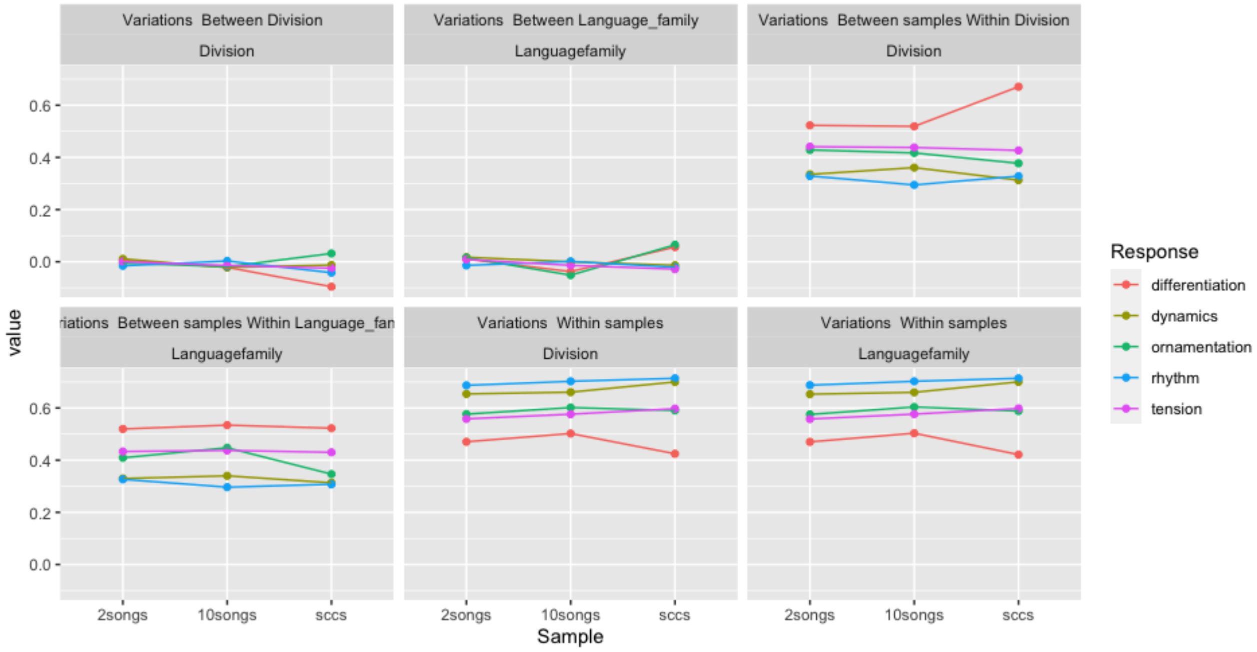

Fig S5. Visual representation of Supplementary Data S1. AMOVA results showing the response, variance partition, dataset, macrogrouping, and variance explained. The test is performed for all response variables, across the 2 song or more (5,242 songs and 719 societies), 10 song or more (3,039 songs and 220 societies), or SCCS dataset (724 songs and 110 societies).

S4.2 Comparison of F_{ST} and Phi_{ST} distributions

To contextualize the size of between-society variation, we compare genetic distances (expressed as F_{ST}), with a musical distance measure (Phi_{ST}, an analogous and comparable statistic to F_{ST} (38); Figure S3). Both Phi_{ST} and F_{ST} are corresponding measurements for the fraction of variance that is not shared between a pair of populations, where 1 indicates no shared variation and 0 indicates complete overlap in variation. Genetic F_{ST} values in our sample range between 0 (complete overlap of genetic diversity) and 0.33 (33% of variation is shared between populations), with a median of 0.1 - similar to other global genetic surveys (40, 41). Musical Phi_{ST} values range between complete overlap (Phi_{ST} = 0), and complete dissimilarity (Phi_{ST} = 1). Median musical Phi_{ST} values range between 0.18 and 0.48, while again observing a division between dimensions that vary between-societies (Articulation, Ornamentation, and Tension range: 0.39 - 0.48) and those that vary more within-societies (Rhythm = 0.29 and Dynamics = 0.18; Table S10). On average, between-society musical differences are between two and four times higher than genetic distances, although variation in musical distances is also considerably higher than genetic variation.

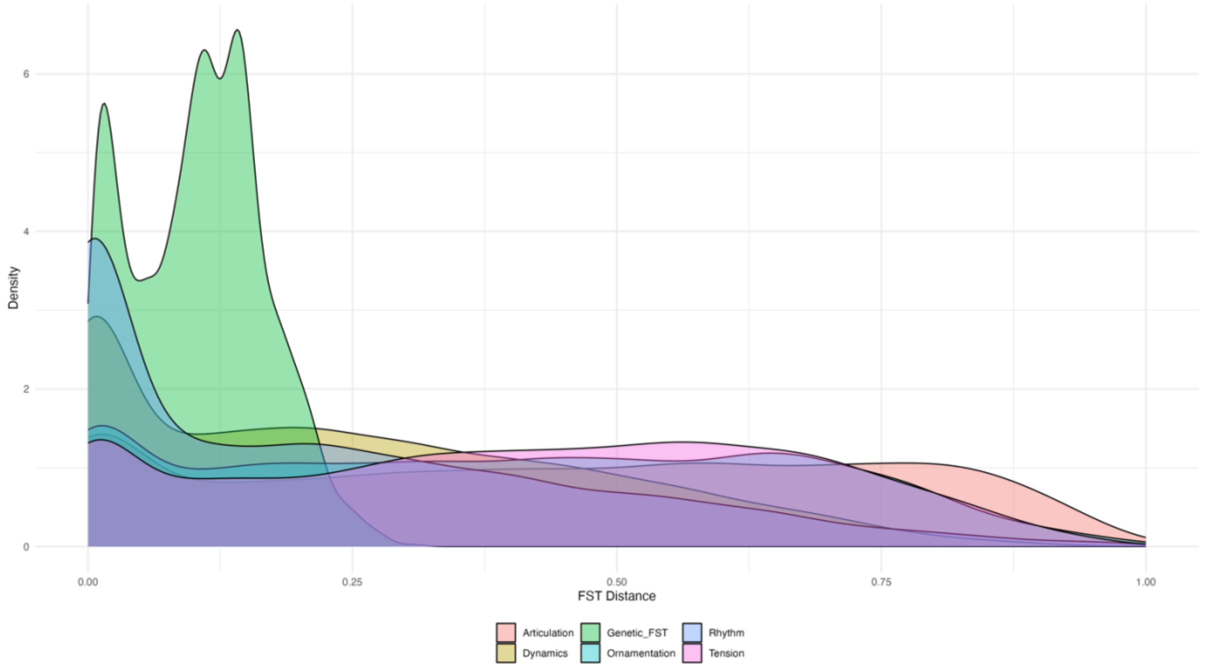

Fig. S6: Density plot of Musical Phi_{ST} and Genetic F_{ST} values (N = 117). While Genetic F_{ST} values vary between 0 and 0.25, Musical similarity ranges across the Phi_{ST} 0 - 1 range.

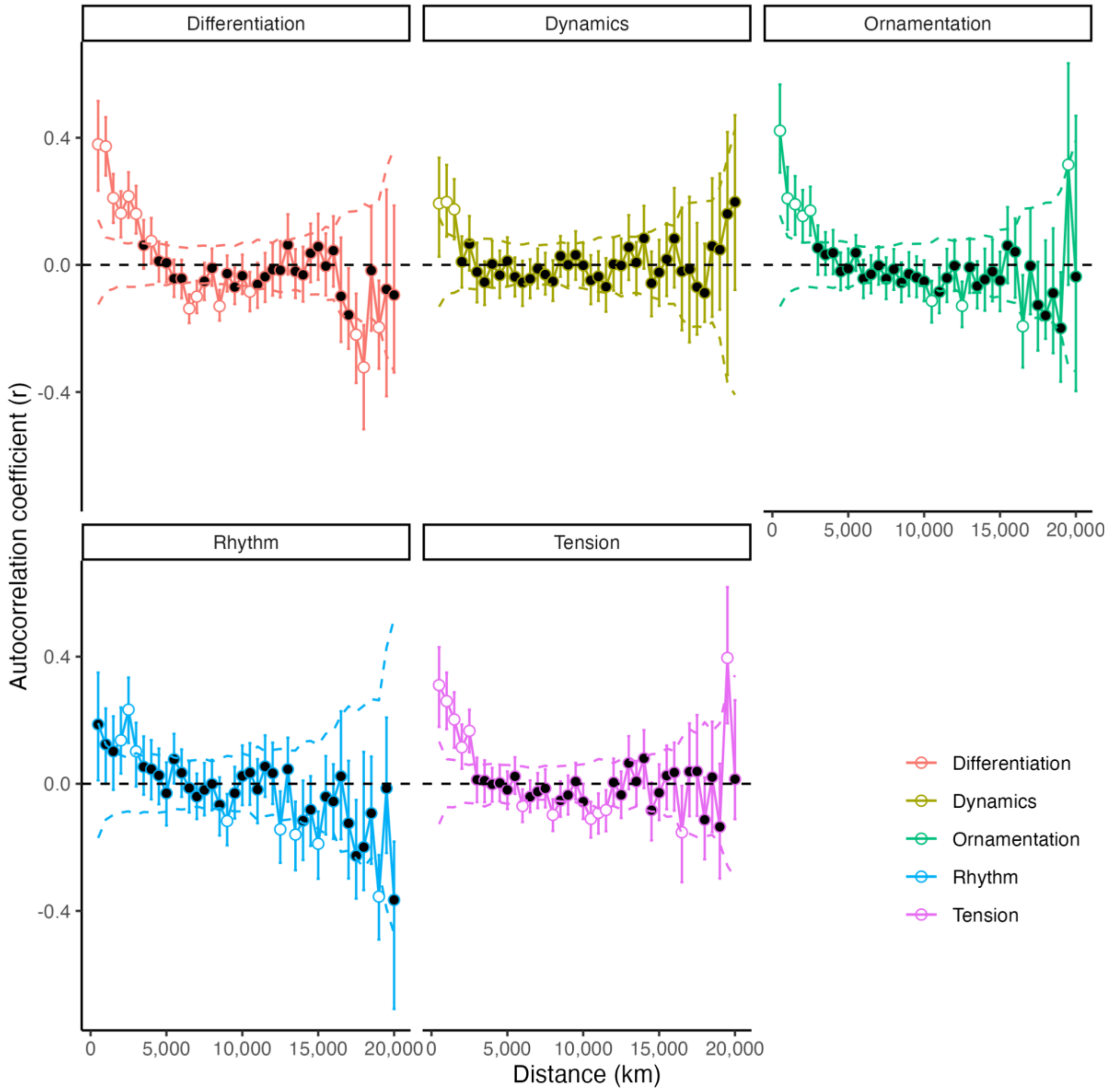

Fig S7. Variogram of each musical dimension, as Musical Phi$_{ST}$ distances, derived from the latent variable analysis for the 2 song or more dataset (N = 117 societies). Autocorrelation (*r*) is shown on the y-axis, with correlations measured at 500km intervals. White shapes indicate significant autocorrelation and black shapes indicate non-significant autocorrelation. Error bars show the 95% confidence intervals for each distance.

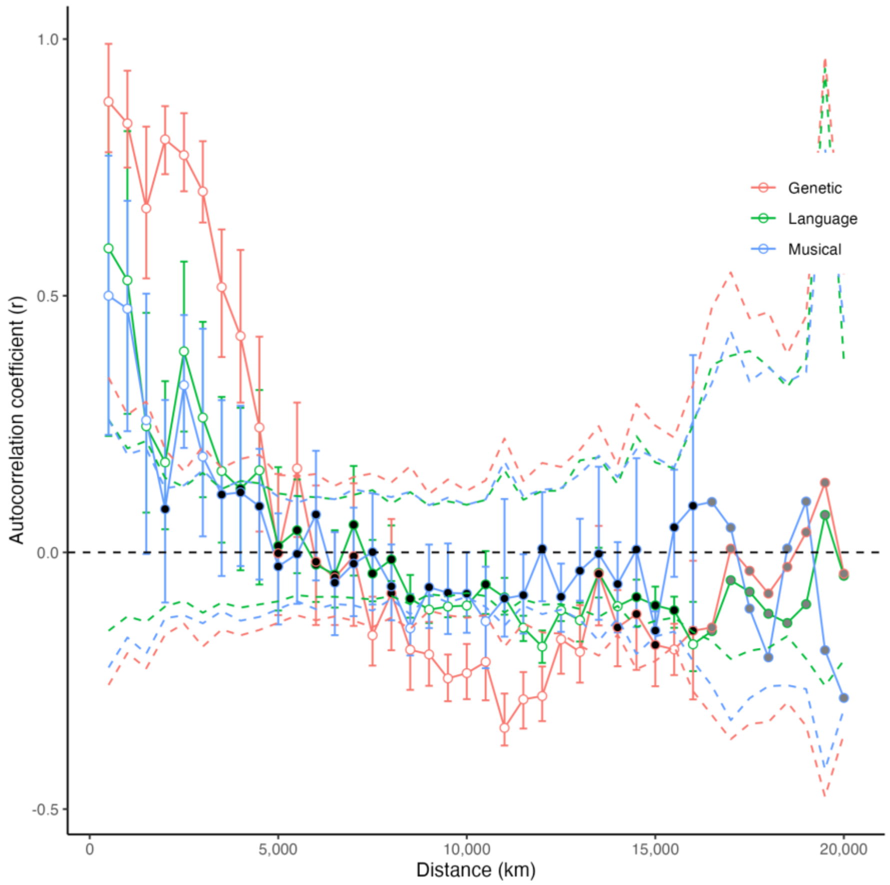

Fig S8. Variogram of Genetic F_{ST} distances, phylogenetic distance from the global language phylogeny, and Musical Phi_{ST} distances for the 10 song or more dataset (N = 44 societies). Autocorrelation (*r*) is shown on the y-axis, with correlations measured at 500km intervals. White shapes indicate significant autocorrelation and black shapes indicate non-significant autocorrelation. Error bars show the 95% confidence intervals for each distance.

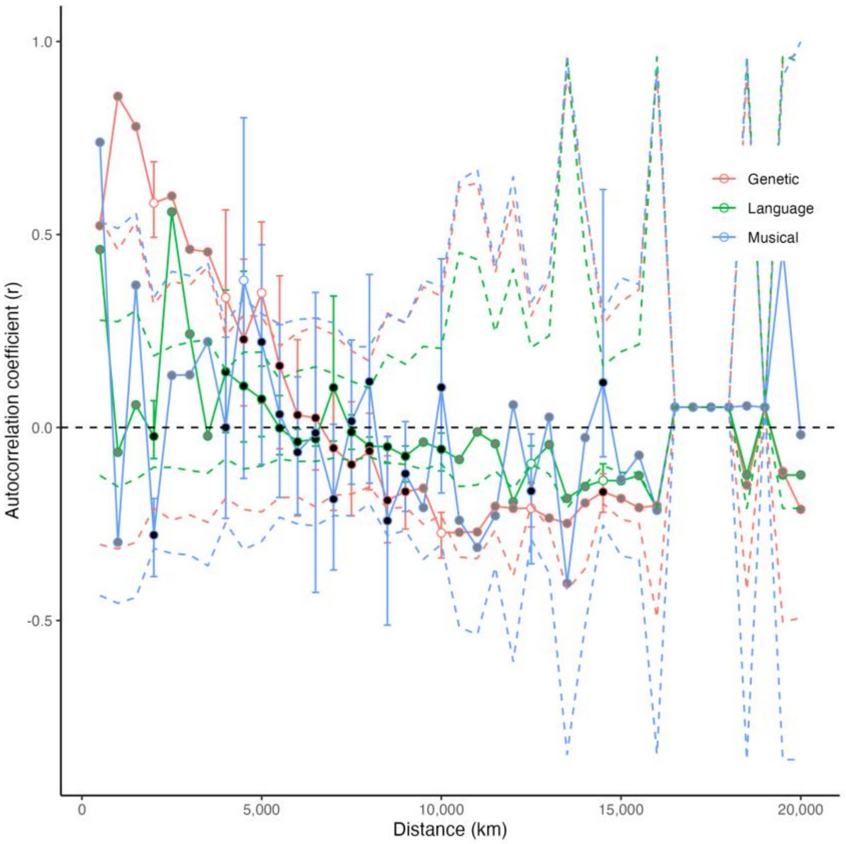

Fig S9. Variogram of Genetic F_{ST} distances, phylogenetic distance from the global language phylogeny, and Musical Phi_{ST} distances for the SCCS (N = 22 societies). Autocorrelation (*r*) is shown on the y-axis, with correlations measured at 500km intervals. White shapes indicate significant autocorrelation and black shapes indicate non-significant autocorrelation. Error bars show the 95% confidence intervals for each distance.

Table S11. Delta scores for three regional subsets of 50 societies: Africa, Oceania, and Europe. For three datasets: 2 songs or more, 10 songs or more, and the SCCS sample.

	**Africa**	**Oceania**	**Europe**	**Dataset**
Articulation	0.26	0.30	0.32	2 Songs
Dynamics	0.34	0.36	0.37	2 Songs
Ornamentation	0.35	0.38	0.36	2 Songs
Rhythm	0.34	0.35	0.31	2 Songs
Tension	0.35	0.40	0.36	2 Songs
Articulation	0.33	0.21	0.31	10 Songs
Dynamics	0.34	0.32	0.36	10 Songs
Ornamentation	0.35	0.34	0.24	10 Songs
Rhythm	0.34	0.28	0.32	10 Songs
Tension	0.31	0.33	0.35	10 Songs
Articulation	0.26	0.30	0.32	SCCS
Dynamics	0.34	0.36	0.37	SCCS
Ornamentation	0.35	0.38	0.36	SCCS
Rhythm	0.34	0.35	0.31	SCCS
Tension	0.35	0.40	0.36	SCCS

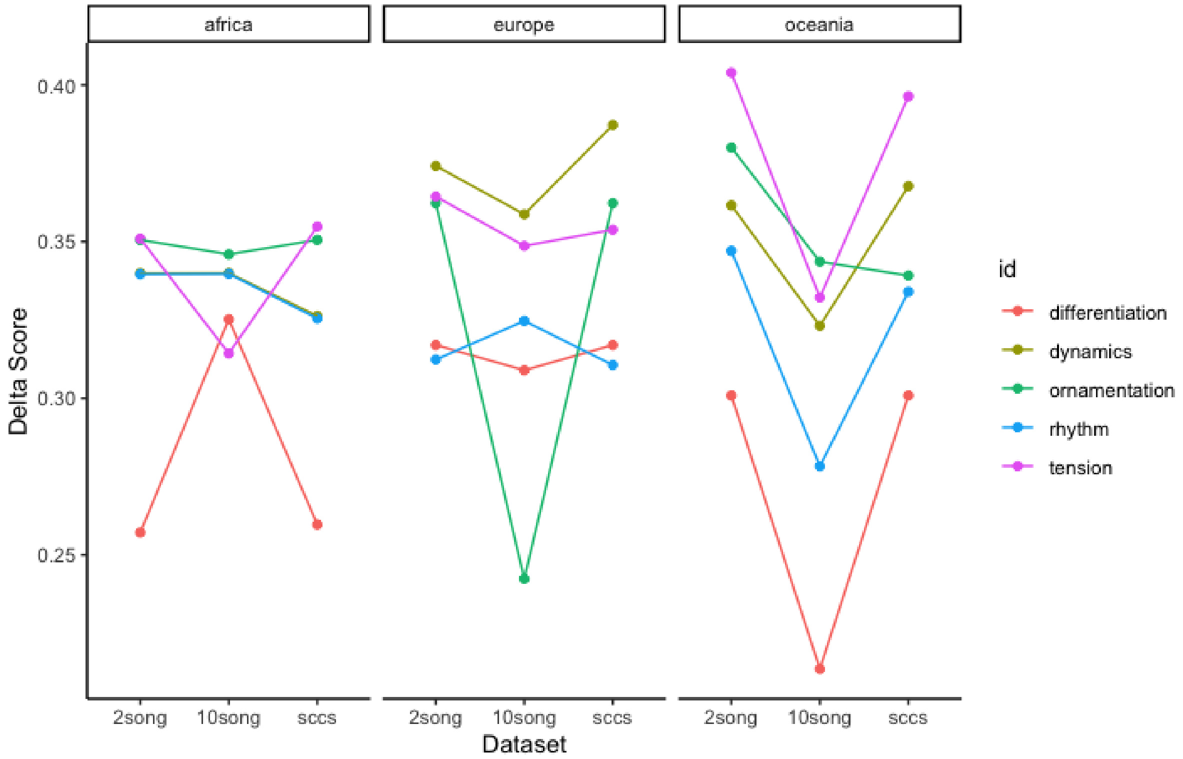

Fig S10. Visualization of table S11. Delta scores for three regional subsets of 50 societies: Africa, Oceania, and Europe. For three datasets: 2 songs or more, 10 songs or more, and the SCCS sample.

Table S12. Pearson Correlation of Partial RDA R2 results across the three datasets. The 2 songs or more dataset contains 5,242 songs and 719 societies, the 10 songs or more dataset contains 3,039 songs and 220 societies, and the SCCS dataset contains 724 songs and 110 societies. The N value for each comparison is the minimum N of the two datasets being compared.

**RDA type**	**Dataset 1**	**Dataset 2**	**Correlation**
3-way	2song	10song	0.81
3-way	2song	SCCS	0.03
3-way	10song	SCCS	0.06

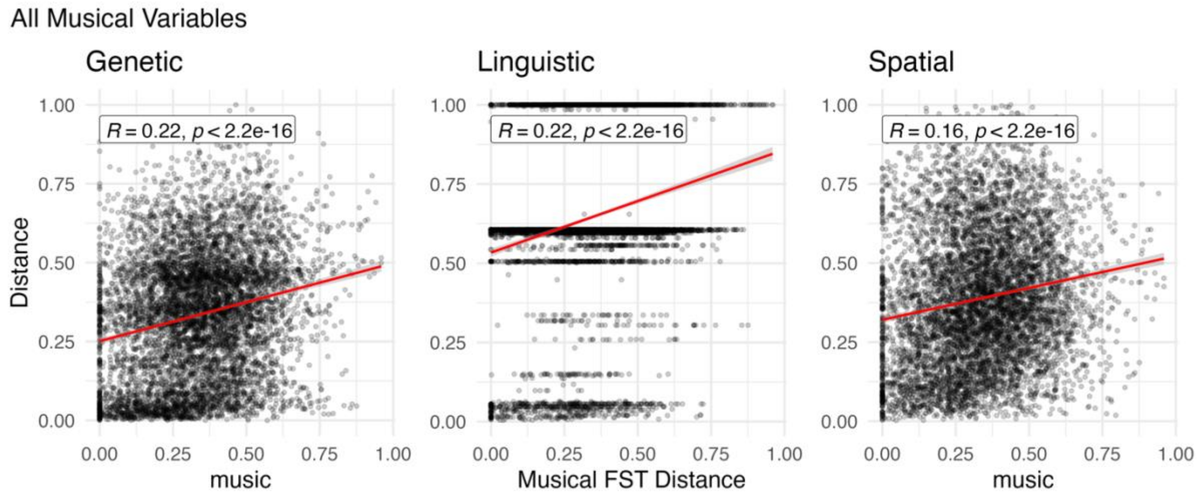

Fig S11. Pairwise plots between a Phi_{ST} matric of all cantometrics variables, and genetic, linguistic, and spatial distance. Linear regression line shown in red with Pearson's R value in the top right. Plots show distances between pairs of 117 societies (N = 6,786).

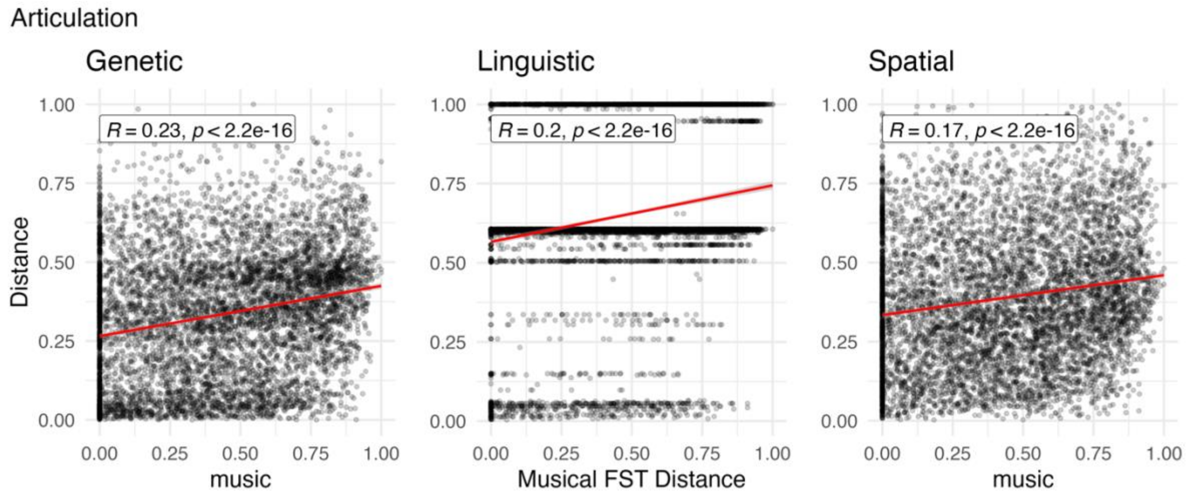

Fig S12. Pairwise plots between a Phi_{ST} matrix of Articulation, and genetic, linguistic, and spatial distance. Linear regression line shown in red with Pearson's R value in the top right. Plots show distances between pairs of 117 societies (N = 6,786).

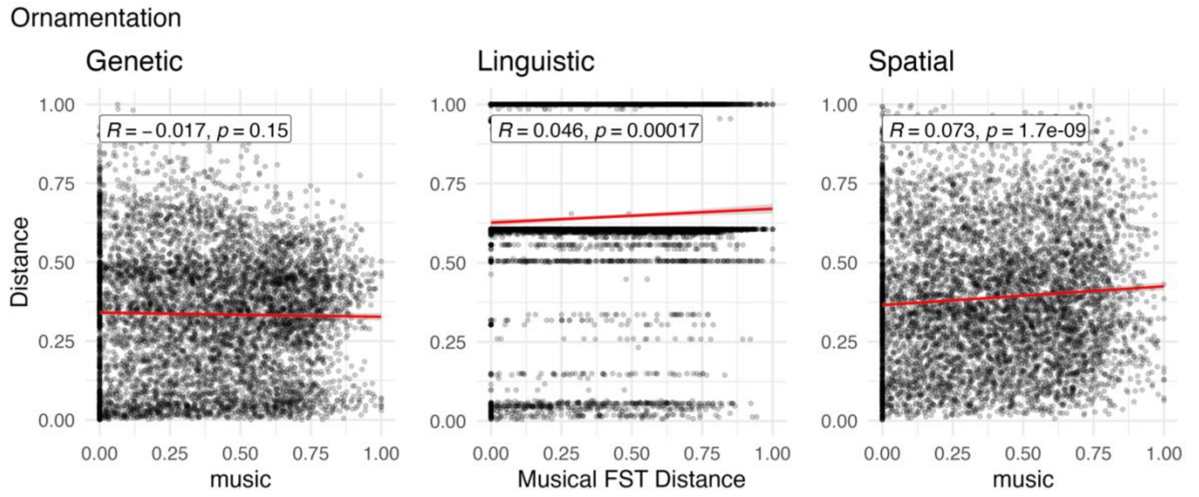

Fig S13. Pairwise plots between a Phi_{ST} matrix of Ornamentation, and genetic, linguistic, and spatial distance. Linear regression line shown in red with Pearson's R value in the top right. Plots show distances between pairs of 117 societies (N = 6,786).

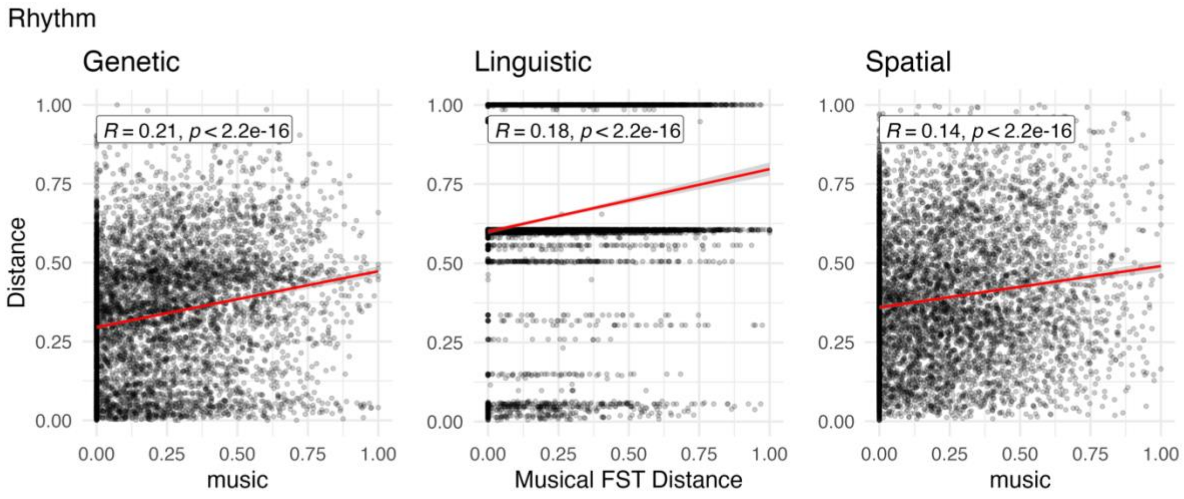

Fig S14. Pairwise plots between a Phi_{ST} matrix of Rhythm, and genetic, linguistic, and spatial distance. Linear regression line shown in red with Pearson's R value in the top right. Plots show distances between pairs of 117 societies (N = 6,786).

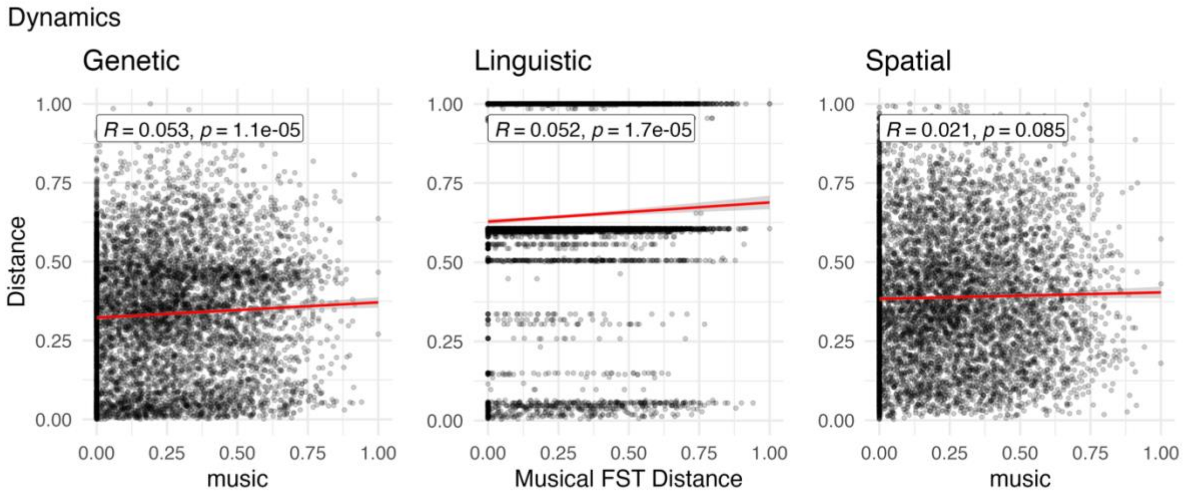

Fig S15. Pairwise plots between a Phi_{ST} matrix of Dynamics, and genetic, linguistic, and spatial distance. Linear regression line shown in red with Pearson's R value in the top right. Plots show distances between pairs of 117 societies (N = 6,786).

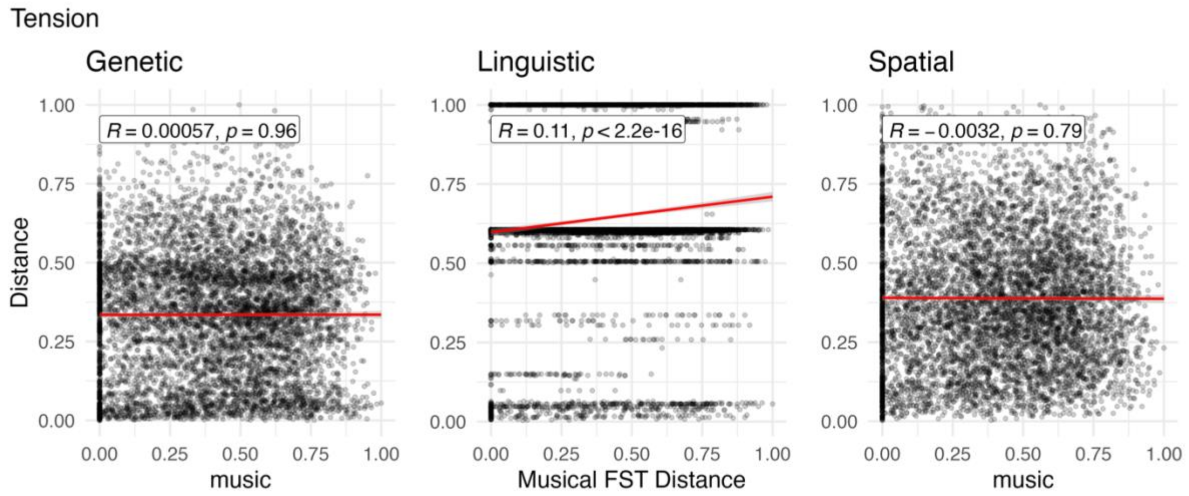

Fig S16. Pairwise plots between a Phi_{ST} matrix of Tension, and genetic, linguistic, and spatial distance. Linear regression line shown in red with Pearson's R value in the top right. Plots show distances between pairs of 117 societies (N = 6,786).

References

1. Wood, A. L. C. *et al.* The Global Jukebox: A public database of performing arts and culture. *PLOS ONE* **17**, e0275469 (2022).
2. Forkel, R. & Hammarström, H. Glottocodes: Identifiers linking families, languages and dialects to comprehensive reference information. *Semantic Web* **13**, 917–924 (2022).
3. Barbieri, C. *et al.* A global analysis of matches and mismatches between human genetic and linguistic histories. *Proceedings of the National Academy of Sciences* **119**, e2122084119 (2022).
4. R Core Team. *R: A Language and Environment for Statistical Computing*. https://www.R-project.org/ (2021).
5. Lomax, A. Factors of Musical Style. in *In Theory and Practice: Essays presented to Gene Weltfish.* (ed. Diamond, S.) (Mouton, The Hague, 1980).
6. Dray, S. & Dufour, A.-B. The ade4 package: implementing the duality diagram for ecologists. *Journal of statistical software* **22**, 1–20 (2007).
7. Aktas, C. *Haplotypes: Manipulating DNA Sequences and Estimating Unambiguous Haplotype Network with Statistical Parsimony*. https://CRAN.R-project.org/package=haplotypes (2020).
