# Supplementary material for: Global musical diversity is largely independent of linguistic and genetic histories
Source: Nat Commun. 2024 May 10;15:3964. doi: 10.1038/s41467-024-48113-7 (PMC11087526; doi:10.1038/s41467-024-48113-7)
Supplement: Supplementary file 3 — Description of Additional Supplementary Files [file 41467_2024_48113_MOESM3_ESM.pdf]

Description of Additional Supplementary Files

File name: Supplementary Data 1
Description: AMOVA results showing the response, variance partition, dataset, macrogrouping, and variance explained. The test is performed for all response variables, across the 2 song or more (5,242 songs and 719 societies), 10 song or more (3,039 songs and 220 societies), or SCCS dataset (724 songs and 110 societies).

File name: Supplementary Data 2
Description: Autocorrelation values for linguistic similarity calculated at 500km intervals between 500km and 20,000km. r is the correlation value. r, U and L values are adjusted by the correction factor. Uncorrected values are shown as r uc, U uc, L uc. Bootstrap mean, Ur, Lr are also adjusted by the correction factor. Upper (Ur error) and lower (Lr error) error bars bound the 95% confidence interval about r as determined by bootstrap resampling. Upper (U) and lower (L) confidence limits bound the 95% confidence interval about the null hypothesis of No spatial structure for the combined data set as determined by permutation.

File name: Supplementary Data 3
Description: Autocorrelation values for genetic similarity calculated at 500km intervals between 500km and 20,000km. r is the correlation value. r, U and L values are adjusted by the correction factor. Uncorrected values are shown as r uc, U uc, L uc. Bootstrap mean, Ur, Lr are also adjusted by the correction factor. Upper (Ur error) and lower (Lr error) error bars bound the 95% confidence interval about r as determined by bootstrap resampling. Upper (U) and lower (L) confidence limits bound the 95% confidence interval about the null hypothesis of No spatial structure for the combined data set as determined by permutation.

File name: Supplementary Data 4
Description: Autocorrelation values for musical similarity calculated at 500km intervals between 500km and 20,000km. r is the correlation value. r, U and L values are adjusted by the correction factor. Uncorrected values are shown as r uc, U uc, L uc. Bootstrap mean, Ur, Lr are also adjusted by the correction factor. Upper (Ur error) and lower (Lr error) error bars bound the 95% confidence interval about r as determined by bootstrap resampling. Upper (U) and lower (L) confidence limits bound the 95% confidence interval about the null hypothesis of No spatial structure for the combined data set as determined by permutation.

File name: Supplementary Data 5
Description: List of genetic sources which provide the data used for FST calculations. Each citation is followed by the populations it contributed, including the linked glottocode, and number of individuals analyzed from that population. This information can also be found indexed by population in the Github repository.

File name: Supplementary Data 6
Description: Results for Partial Mantel and Partial RDA analysis with societies holding 2 or more songs (5,242 songs and 719 societies). Mantel test p-values are two-sided tests. Z-values are calculated following Matsumae et al. (2020). The final column shows probability values that the true R2 is more than 1 standard deviation away from the permuted R2. Values greater than 0.95 are considered significant.

File name: Supplementary Data 7
Description: Results for Partial Mantel and Partial RDA analysis with societies holding 10 or more songs (3,039 songs and 220 societies).

File name: Supplementary Data 8
Description: Results for Partial Mantel and Partial RDA analysis with societies within the SCCS sample (724 songs and 110 societies).
