# Supplementary material for: Global musical diversity is largely independent of linguistic and genetic histories
Source: Nat Commun. 2024 May 10;15:3964. doi: 10.1038/s41467-024-48113-7 (PMC11087526; doi:10.1038/s41467-024-48113-7)
Supplement: Supplementary file 5 — Reporting Summary [file 41467_2024_48113_MOESM5_ESM.pdf]

nature portfolio

Corresponding author(s): Sam Passmore

Last updated by author(s): 18 July

Reporting Summary

Nature Portfolio wishes to improve the reproducibility of the work that we publish. This form provides structure for consistency and transparency in reporting. For further information on Nature Portfolio policies, see our Editorial Policies and the Editorial Policy Checklist.

Statistics

For all statistical analyses, confirm that the following items are present in the figure legend, table legend, main text, or Methods section.

n/a	Confirmed	
☐	☒	The exact sample size (*n*) for each experimental group/condition, given as a discrete number and unit of measurement
☐	☒	A statement on whether measurements were taken from distinct samples or whether the same sample was measured repeatedly
☐	☒	The statistical test(s) used AND whether they are one- or two-sided<br>*Only common tests should be described solely by name; describe more complex techniques in the Methods section.*
☐	☒	A description of all covariates tested
☐	☒	A description of any assumptions or corrections, such as tests of normality and adjustment for multiple comparisons
☐	☒	A full description of the statistical parameters including central tendency (e.g. means) or other basic estimates (e.g. regression coefficient) AND variation (e.g. standard deviation) or associated estimates of uncertainty (e.g. confidence intervals)
☐	☒	For null hypothesis testing, the test statistic (e.g. *F*, *t*, *r*) with confidence intervals, effect sizes, degrees of freedom and *P* value noted<br>*Give P values as exact values whenever suitable.*
☒	☐	For Bayesian analysis, information on the choice of priors and Markov chain Monte Carlo settings
☒	☐	For hierarchical and complex designs, identification of the appropriate level for tests and full reporting of outcomes
☐	☒	Estimates of effect sizes (e.g. Cohen's *d*, Pearson's *r*), indicating how they were calculated

Our web collection on statistics for biologists contains articles on many of the points above.

Software and code

Policy information about availability of computer code

Data collection	We combine three datasets: The Global Jukebox (Wood et. al 2022; https://zenodo.org/records/4895999) GeLaTo (Barbieri et al. 2022; https://zenodo.org/records/7233267), and The Global Language Phylogeny (Bouckaert et al, 2023; https://osf.io/yzxv9/).
Data analysis	All code for this project is archived at https://zenodo.org/records/10817212. Analysis is performed in R and Excel. We use the following packages for core analyses in R: lavaan (Rossell, 2012); haplotypes (Aktas, 2020); vegan (Oksanen et a. 2013). We use the Genalex add-in within Excel (Peakall & Smouse, 2006).

For manuscripts utilizing custom algorithms or software that are central to the research but not yet described in published literature, software must be made available to editors and reviewers. We strongly encourage code deposition in a community repository (e.g. GitHub). See the Nature Portfolio guidelines for submitting code & software for further information.

Data

Policy information about availability of data

All manuscripts must include a data availability statement. This statement should provide the following information, where applicable:

- Accession codes, unique identifiers, or web links for publicly available datasets
- A description of any restrictions on data availability
- For clinical datasets or third party data, please ensure that the statement adheres to our policy

All data processed and used in this study are accessible at https://zenodo.org/records/10817212. All data is freely accessible. The processed data are available at

the same address within the folder 'processed_data'. Some results and data processing take significant computing time, so we keep pre-computed results in the same repository and folder. This project only utilises existing datasets. The original sources of the data are as follows: The Global Jukebox41, with data accessible from https://github.com/theglobaljukebox/cantometrics; GeLaTo12, with data accessible from https://github.com/gelato-org/gelato-data, additionally, the source of the population samples used are also listed in Table S15; The global language phylogeny40, with data accessible at https://osf.io/yzxv9/. To listen to the audio, and read more detail on the Cantometric coding scheme visit http://theglobaljukebox.org. Please cite 41 if using Cantometrics, or other Global Jukebox data. Global Jukebox datasets are archived with ZENODO, and the DOI provided by ZENODO should be used when citing releases of Global Jukebox datasets, which are available within the GitHub organization.

Research involving human participants, their data, or biological material

Policy information about studies with human participants or human data. See also policy information about sex, gender (identity/presentation), and sexual orientation and race, ethnicity and racism.

Reporting on sex and gender	NA
Reporting on race, ethnicity, or other socially relevant groupings	NA
Population characteristics	NA
Recruitment	NA
Ethics oversight	NA

Note that full information on the approval of the study protocol must also be provided in the manuscript.

Field-specific reporting

Please select the one below that is the best fit for your research. If you are not sure, read the appropriate sections before making your selection.

☐ Life sciences ☒ Behavioural & social sciences ☐ Ecological, evolutionary & environmental sciences

For a reference copy of the document with all sections, see nature.com/documents/nr-reporting-summary-flat.pdf

Behavioural & social sciences study design

All studies must disclose on these points even when the disclosure is negative.

Study description	Quantitative and exploratory analysis
Research sample	The Global Jukebox contains Cantometric codings for 5,776 songs from 1,026 societies on 37 different variables (Wood et al., 2022). The complete dataset used here contains 5,242 songs from the 719 societies represented by at least two songs.
Sampling strategy	For the main analyses, we use the largest sample size available, which represents a convenience sample. To assess the robustness of our conclusions we sub-sample using three strategies: Using all societies who have two or more songs recorded in the Global Jukebox. All societies who have ten or more songs societies recorded in the Global Jukebox. Using all societies who have two or more songs recorded and are part of the Standard Cross Cultural Sample. We do not perform a sample size a priori, instead using the full breadth of the dataset available to make the largest musical-linguistic-genetic comparison in the literature.
Data collection	Data was not collected for this study. We only use secondary datasets.
Timing	Songs within the Global Jukebox were primarily collected from the 1940s to 1980s, with a maximum range of recording dates between 1904 to 1982.
Data exclusions	Songs were excluded from the analyses if they was only one song recorded for that group.
Non-participation	No Participants were used in this study
Randomization	This study relies on observational data from societies across the globe. To maximize the breadth of societies compared, we do not randomly sub-sample our dataset.

Reporting for specific materials, systems and methods

We require information from authors about some types of materials, experimental systems and methods used in many studies. Here, indicate whether each material, system or method listed is relevant to your study. If you are not sure if a list item applies to your research, read the appropriate section before selecting a response.

Materials & experimental systems

n/a	Involved in the study
☒	☐ Antibodies
☒	☐ Eukaryotic cell lines
☒	☐ Palaeontology and archaeology
☒	☐ Animals and other organisms
☒	☐ Clinical data
☒	☐ Dual use research of concern
☒	☐ Plants

Methods

n/a	Involved in the study
☒	☐ ChIP-seq
☒	☐ Flow cytometry
☒	☐ MRI-based neuroimaging
